# Estimating the structure factors in X-ray diffraction

**DOI:** 10.1107/S2053273318007593

**Published:** 2018-08-08

**Authors:** Paul F. Fewster

**Affiliations:** a Brighton, UK

**Keywords:** structure factors, imperfect crystals, diffraction theory, serial crystallography, powder diffraction

## Abstract

This article takes the concepts of the ‘new diffraction theory’ [Fewster (2014 ▸). *Acta Cryst.* A**70**, 257–282] and examines the implications for the interpretation of experimental results and the estimation of structure factors. Further experimental evidence is included to justify the conclusions in the theory, showing that the residual intensity at twice the Bragg angle is a diffraction effect and not associated with the crystal shape. This ‘enhancement’ effect is independent of whether kinematical or dynamical theories are applied and can lead to a clearer understanding of how the dynamical effects are suppressed in imperfect crystals. By applying the idea that the higher-order peaks are due to path lengths of *n*λ, it is shown that ‘systematically absent’ reflections in the conventional theory may not be absent. Because this new theory considers the intensity to be more distributed, it suggests that the entire structure factor can be difficult to capture by experiment. This article suggests some routes to achieve a good approximation of the structure factors for typical methods of data collection. Any measurement of intensity with background removal will exclude some of the distributed intensity, again leading to an underestimate of the structure factors, and therefore the missing intensity needs to be estimated.

## Abstract 2

The meaning of the structure factor and how it impacts on the determination of structures are reassessed. A route to obtaining the structure factors is presented for several data collection methods and crystal qualities.

## 1. Introduction   

X-ray diffraction analysis has relied to an increasing extent on the accuracy of intensity measurements to reveal important structural information in complex molecules, *e.g.* functional groups in proteins *etc*. It is therefore crucial to ensure that the derived structural model closely resembles reality, which can only be achieved if the description of diffraction is sufficiently complete. Also, the reliability of the measured data can only be estimated with confidence if a complete interpretation of the diffraction pattern is available.

The most complete description of diffraction should include all the scattered amplitudes from all the atoms at all detection positions, including atomic vibrations and re-scattering. All the amplitudes will be coherently related which makes the inverse problem of estimating the structure from the diffraction pattern a difficult challenge. Also, any unforeseen microstructure features may confuse the interpretation. The dominating features in a diffraction pattern are the strong peaks that Bragg (1913 ▸) associated with specular (mirror) reflections from crystal planes. This explanation assumes that all the atoms aligned in these planes concentrate their amplitudes at one position and can be simply interpreted, or that the amplitude diminishes from the peak due to size effects (James, 1962 ▸; Authier, 2001 ▸) *etc*., which is the shape transform.

Soon after Bragg published his explanation, Darwin (1914 ▸) recognized that the scattering from a set of parallel planes introduces re-scattering from the underside of planes above. This led to the diminution of the incident beam and explained the width of the profile. Ewald (1916 ▸, 1917 ▸) and Laue (1931 ▸) took this further and introduced the polarizability of the electron density by the incoming electromagnetic wave by considering the crystal as a series of dipoles (Ewald) and a distributed electron density (Laue). These two works form the basis of the interaction of the incident wave and the crystal. The electric field vector component parallel to the surface must be continuous which defines the location (2θ) of the scattered intensity for a given incident angle (James, 1962 ▸; Fewster, 2015 ▸ *etc*.). This gives the profile shape and its direction, *e.g.* crystal truncation rods.

There have been many attempts to apply dynamical theory to imperfect crystals. Initially, a perturbation approach to dynamical theory (Penning & Polder, 1961 ▸) was applied but is only relevant to small distortions. Takagi (1962 ▸, 1969 ▸) took the argument further by introducing a locally varying polarizability, which pushed the bounds of dynamical theory closer to modelling less-than-perfect crystals. Kato (1976 ▸) introduced ideas of regions that scatter incoherently, which is analogous to the mosaic crystal model initiated by Darwin (1922 ▸) and pursued further by Zachariasen (1945 ▸). This has become the standard description of an imperfect crystal and the justification for using kinematical theory. Dynamical theory associated with the works above is far from perfect, because it is calculated assuming there are only two significant waves (two-beam dynamical theory), *i.e.* the incident and scattered waves. The introduction of other reflections and surface scattering are ignored. Introducing more beams adds significant complexity (Holý & Fewster, 2003 ▸), but because the conventional approach defines the structure factor for a specific region (or layer in that article) the interfaces are too abrupt which is evident in perfectly periodic multilayer structures. Holý & Fewster (2008 ▸) took the argument further by calculating the scattering from very thin lamellae (0.01 of the unit-cell dimension) and using Fresnel’s equations. The whole profile can be calculated over the whole length of the truncation rod, allowing the introduction of imperfections. Chantler (1992*a* ▸,*b* ▸) has explored dynamical theory of bent perfect and imperfect crystals by splitting the calculation region of the crystal into thin lamellae and blocks which really gives an indication of the complexity required to model the scattering. The first of these articles reviews some of the published approaches to modelling less perfect crystals.

The general assumption in conventional theory is that an imperfect crystal is mosaic, which is also discussed by Authier (2001 ▸) where the defects break the coherence to produce an agglomeration of mosaic blocks scattering incoherently with respect to their neighbours. Each perfect block then scatters dynamically, and because of their small size the dynamical effects are small. The diffraction from each block is weak, has broad features and fringing related to its shape and can be explored by varying the incident angle. Each of these incident angles will give intensity further away from the 2θ_B_ position, *i.e.* the crystal shape adds intensity around but not at 2θ_B_.

**Figure 1:**
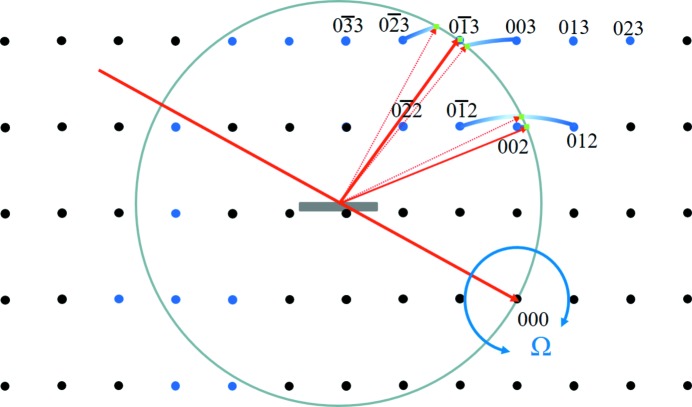
The new theory in terms of the Ewald sphere construction. All the reciprocal-lattice points coloured blue can form intensity for this given incident angle at their respective 2θ_B_ [subject to the conditions contained in Fewster (2014 ▸), *i.e.* 0 < Ω < 2θ_B_]. The distance of the reciprocal-lattice point to the surface of the Ewald sphere along the arc in Ω defines its amplitude. The amplitude decreases as the distance increases, *e.g.* 0
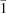
3 is in the Bragg condition and the amplitude is at its maximum value, whereas 002 is weaker and 0
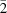
3 is very weak *etc*. The arcs drawn for some of the reflections give a guide to judge the strength of the scattering by the distance along the arc from the Ewald sphere maximum value. We can consider the Ewald sphere surface to have a thickness with a profile given by Fewster [2014 ▸, equation (4)].

The description by Fewster (2014 ▸, 2016 ▸) is different in that a single incident angle will form a full diffraction pattern. For example, a crystal plane rotated away from the Bragg condition will still show a peak at 2θ_B_, but one that is considerably weaker. This applies to all the fringes associated with a peak. This can be understood by imagining the conventional Ewald sphere surface to have a thickness and the ‘conventional’ construction represents the maximum interaction with the reciprocal lattice (in the conventional theory the Ewald sphere surface has no thickness). The surface thickness is represented by a scattering vector (of magnitude 1/*d_hkl_*) being swept around the reciprocal-lattice origin 000 (Fig. 1 ▸). The thickness profile is given by Fig. 15, Appendix *A* [App appa], which is a plot of equation (4*b*) in Fewster (2014 ▸). The final diffraction pattern for this single incident angle is therefore nearly complete, with the intensity being defined by how far the reciprocal-lattice features are from the optimum orientation for that incident angle, *i.e.* the surface of the conventional Ewald sphere. This description explains the existence of a polycrystalline diffraction pattern from very few crystals and the observation of multiple peaks in X-ray free-electron laser (XFEL) diffraction patterns. This is completely independent of the shape, because the associated fringing will be subject to the same description, *i.e.* there will be a tail of intensity along its own 2θ_B_. This full diffraction pattern will be very weak and rarely seen except at very intense sources like an XFEL. This diffraction pattern is observed in simulations of point scatterers (Fewster, 2017 ▸), with the maximum intensity of a feature occurring when it intersects the surface of the conventional Ewald sphere, but it remains visible remote from this condition (Fig. 16 in Appendix *A* [App appa]). A small crystal will have a broad Ewald sphere surface thickness profile, such that the contrast in the pattern will be reduced, making the peaks in the pattern dominate over the fringes, which may be closer to the ‘conventional’ Ewald sphere surface, as in Fig. 16. This is simply a pattern that we would expect in optical diffraction. These arcs of persistent intensity at 2θ_B_ remote from the Bragg condition can be directly observed with laboratory sources and they are covered in the next section. This description removes the necessity to modify the microstructure to ensure some unexplained feature touches the surface of the conventional Ewald sphere.

Now that serial crystallography at synchrotrons and XFELs has come to the fore, there are a few clues that there is something amiss. Perhaps one of the most fascinating is the variability in the measured intensity, which has been termed ‘partiality’ and has been accommodated by additional parameters associated with variations in beam flux, crystal size, spectral dispersion (Kirian *et al.*, 2010 ▸). Other parameters to account for this variability are the inclusion of mosaic spread and a modified Lorentz factor (Kabsch, 2014 ▸). White (2014 ▸) has introduced a nest of Ewald spheres from which the ‘partiality’ can be estimated by the distance of the reciprocal-lattice points to the Ewald sphere. A similar approach has been taken by Uervirojnangkoorn *et al.* (2015 ▸) *etc*. More recently, Wojtas *et al.* (2017 ▸) have explained the observation of several diffraction peaks by partial capture of a reciprocal-lattice point; from this we might ask how does the surface of the Ewald sphere with no thickness capture intensity from a diffraction pattern? Also, in a detailed study of the correlation of the intensity from different reflections Öztürk *et al.* (2015 ▸) have concluded that the scattering from nanocrystals can contribute to several Laue spots simultaneously and to account for this the authors have included a modified Lorentz factor. These additional parameters may accommodate the observations in serial crystallography, but are they a true description? Kroon-Batenburg *et al.* (2015 ▸) have monitored the *R* factor whilst introducing the modified Lorentz factor and ‘partialities’ resulting in better agreement, but it was also recognized that the model parameters associated with partiality still require improvement. More recently Sharma *et al.* (2017 ▸) have proposed a correction for the observed asymmetric distribution of intensities for a given *hkl* captured in serial crystallography. If the observed intensity is purely associated with the Bragg condition, then each intensity contribution will correspond to somewhere on the peak profile and would be symmetrical. The intensity distribution calculated from the new theory is asymmetric because of the high number of weak contributions. The methods to account for the observations are becoming very complex.

A similar story exists in polycrystalline diffraction, where the number of crystals required to provide a reliable data set becomes unrealistic (Smith, 1999 ▸). Earlier attempts to account for the diffraction profile have suggested slack gearing in instruments (Wolff, 1958 ▸) or mosaic crystals to capture all these reflections (Alexander *et al.*, 1948 ▸). These concerns have largely been forgotten, but with improved instrumentation and with the vast range of crystal types being examined these explanations seem even more unlikely.

## 2. The new theory and experimental evidence   

An X-ray plane wave incident on a plane of atoms will create a spherical wave from each atom, and at large radii along a specific direction these waves will appear planar. All the amplitude contributions that travel along a specific direction 2θ will combine and form a resultant amplitude depending on all their relative phase relationships. At the Bragg condition the maximum amplitude from each plane in a stack is in-phase with all the others, resulting in maximum intensity. As the stack of planes is rotated, the phase is no longer optimal, and the amplitude falls but still forms a peak at the specular scattering angle^1^ for the incident wave angle Ω to these planes. This results in the characteristic Bragg peak and fringes. In the conventional theory, there is one scattered wave *k_H_* for each incident wave *k*_0_ that is related to the scattering vector *S* to give the familiar relation *k_H_* = *k*_0_ + *S*, where both *k* vectors have a magnitude 1/λ and *S* has a magnitude of 1/*d_hkl_* at the Bragg angle.^2^ This can be graphically represented by the Ewald sphere with the conclusion that only features touching its surface can form intensity.

**Figure 2:**
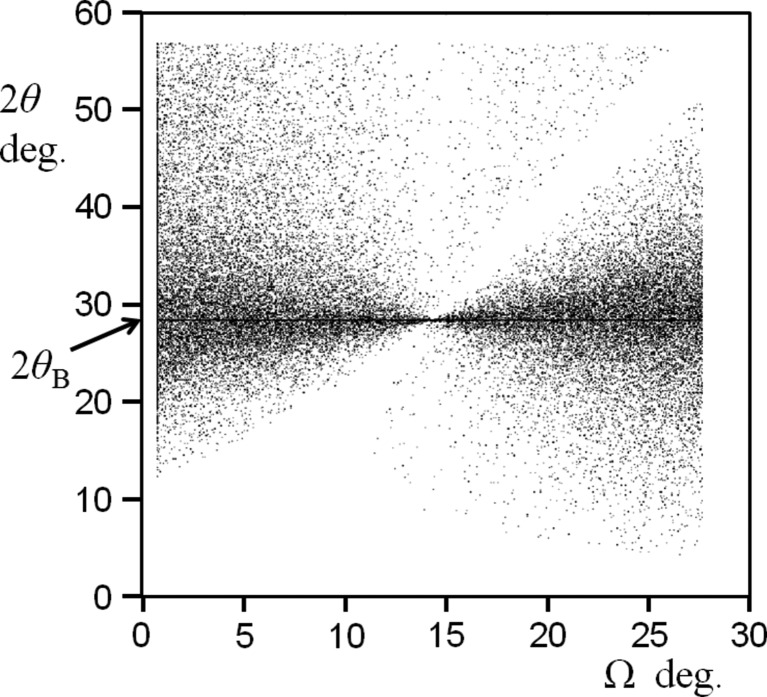
The distribution of path lengths equal to one wavelength (to within a very small tolerance) from scattering points on adjacent planes. As the tolerance is reduced it concentrates on a single value at 2θ_B_ and the other coincidences become sparser. This example calculation uses a crystal plane separation of 0.31356 nm and wavelength of 0.1541 nm, giving rise to a Bragg scattering angle 2θ_B_ = 28.45°.

As shown by Fewster (2016 ▸), a simple structure that would be expected to form a Bragg peak and a crystal truncation rod forms two peaks for an incident angle remote from the Bragg condition: one at the truncation rod intersection and the second at 2θ_B_ for the substrate. As the planes are rotated the intensity associated with the intersection of the truncation rod moves as expected (the specular peak) but the peak at 2θ_B_ remains stationary. The explanation given by Fewster (2014 ▸, 2016 ▸, 2017 ▸) is that there is persistent intensity along 2θ_B_ for the feature of interest, which can be understood by considering the phase relationships between scattering points along each plane and on adjacent planes. For a given incident angle Ω in the specular condition but not at the Bragg condition, *e.g.* on the crystal truncation rod, all points along a single plane are scattering in-phase but they are not in perfect phase with the planes below. If the detector is moved to a new position where 2θ ≠ 2θ_s_, then the points along each plane no longer scatter in perfect phase towards this new 2θ position. This new amplitude from each plane is *A*_Ω_. The path length difference of scattering from points on adjacent planes is also altered by changing 2θ. For example, an incident wave below the Bragg angle will give a weakened intensity at the specular angle (*e.g.* on the crystal truncation rod) and the path length difference is always < λ; if the detector angle 2θ is increased the path length can be increased up to λ. At this position all the planes scatter in-phase to give an amplitude of *NA*_Ω_. Similarly, an incident angle greater than the Bragg angle will have a path length difference > λ at the specular angle and by reducing the detector angle 2θ the planes can be brought back into phase to give an amplitude of *NA*_Ω_. The 2θ when all the planes are brought into phase is defined by *d* and λ for each incident angle Ω. The 2θ angles where the path length is λ, for a given *d*, are plotted in Fig. 2 ▸ for all possible incident angles Ω, based on Fewster (2016 ▸). This is a numerical calculation that has a path length tolerance which is reduced to home-in on the angles and positions that are satisfied exactly for all incident angles. The scattering angles that satisfy this condition always exist at 2θ_B_, whilst the distribution of points elsewhere becomes sparser and more random as the tolerance reduces.

There are good reasons to assume this enhancement at 2θ_B_ occurs in polycrystalline diffraction experiments, for example the data presented in Fewster (2014 ▸) which are reproduced in Fig. 17 of Appendix *A* [App appa]. In that example the probability of observing a single Bragg peak in the conventional theory from this small sample is 1 in 100 000, and of observing three peaks is therefore 1 in 10^15^. If we assumed that the crystals were imperfect and that the conventional theory was correct then we would require 100 000 mosaic blocks in each 3.5 µm crystal, and the blocks would be ∼0.075 µm in size with an intrinsic diffraction width of ∼0.11°. This width is 4× that measured for this standard sample (Fewster & Trout, 2013 ▸).^3^ These requirements make the conventional theory explanation very unlikely. The diffraction width (along 2θ) has been measured to be 0.002° for a ∼10 µm Si crystal isolated (in diffraction space) from a polycrystalline sample (Fewster, 2014 ▸), *i.e.* there is no evidence of mosaicity. The new theory predicts intensity at several diffraction peaks simultaneous with varying intensity (Fig. 1 ▸) and does not require vast numbers of crystal orientations. Each contribution may be small but as the number of crystals increases the sum can lead to a measurable diffraction pattern, with the very rare intensity spike when the Bragg condition is encountered (Fig. 17, Fewster 2014 ▸; Fig. 18 and Fig. 13, Fewster & Andrew, 1999 ▸).

**Figure 3:**
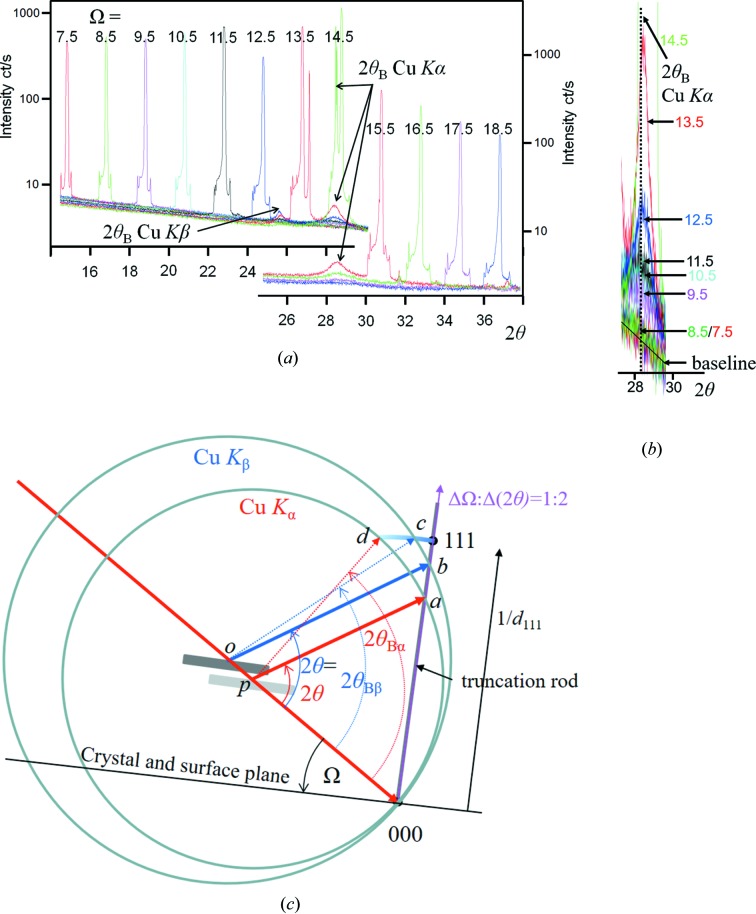
(*a*) Several 2θ scans for fixed Ω settings with the interpretation in (*c*) based on the Ewald sphere construction. The spheres have different radii: 1/λ_α_ and 1/λ_β_, centred on *p* and *o*, respectively. Consider the 2θ scan for Ω = 12.5° in (*a*) (the crystal is orientated 1.7° from the Bragg angle θ_Bα_ for the Cu *K*α wavelength). There is a single specular peak (the intersection of the 2θ scan and the truncation rod) that is described in (*b*), where the specular contributions occur at the same 2θ but capture different positions on the truncation rod *a* and *b*, which is the same for both conventional and new theories. The two peaks corresponding to the *d*_111_ plane spacing for both the Cu *K*α and Cu *K*β wavelengths, *i.e.* at 2θ_α_ and 2θ_β_, should not exist according to the conventional description. The peaks at *c* and *d* can only be described with the new theory. These enhancement peaks can be observed up to |Ω − θ_B_| ≃ 6°, which are given in (*b*) where a baseline for the intensity level from either side of each peak reveals more intensity above the line than below. This is very close to the observational limit for this experiment. The specular peaks are sharp (they are dominated by the proportion of the incident-beam divergence that satisfies this condition, *i.e.* a small region on the sample), and the enhancement peaks are broad (because all the incident-beam divergence directions will form intensity at 2θ_B_ and these exist over the full footprint of the beam on the sample. As the Bragg condition is approached the peak will sharpen because the strongest contributions come from a smaller range of divergence and smaller positions on the sample, and dominate).

This persistent intensity at 2θ_B_ can be observed directly. A larger perfect crystal 〈111〉 orientated wafer of Si is orientated away from the 111 Bragg condition. The incident angle was set to various values, in the range −7° < (Ω − θ_B_) < +4° and the detector was scanned across a large range to include 2θ_B_ (Fig. 3 ▸ *a*). Intensity peaks are observed when 2θ equals 2Ω and when 2θ equals 2θ_B_ for both the Cu *K*α and Cu *K*β wavelengths, *e.g.* when Ω = 12.5° where (Ω − θ_B_) ≃ −1.7°. Peaks at 2θ_B_ for the Cu *K*α wavelength can still be observed at 6° from the Bragg angle. The sharp peaks at 2Ω correspond to the intersection of the Ewald sphere and the crystal truncation rod (predicted by the conventional theory) which comes to a maximum at the Bragg condition (Ω = θ_B_ ≃ 14.2°).

**Figure 4:**
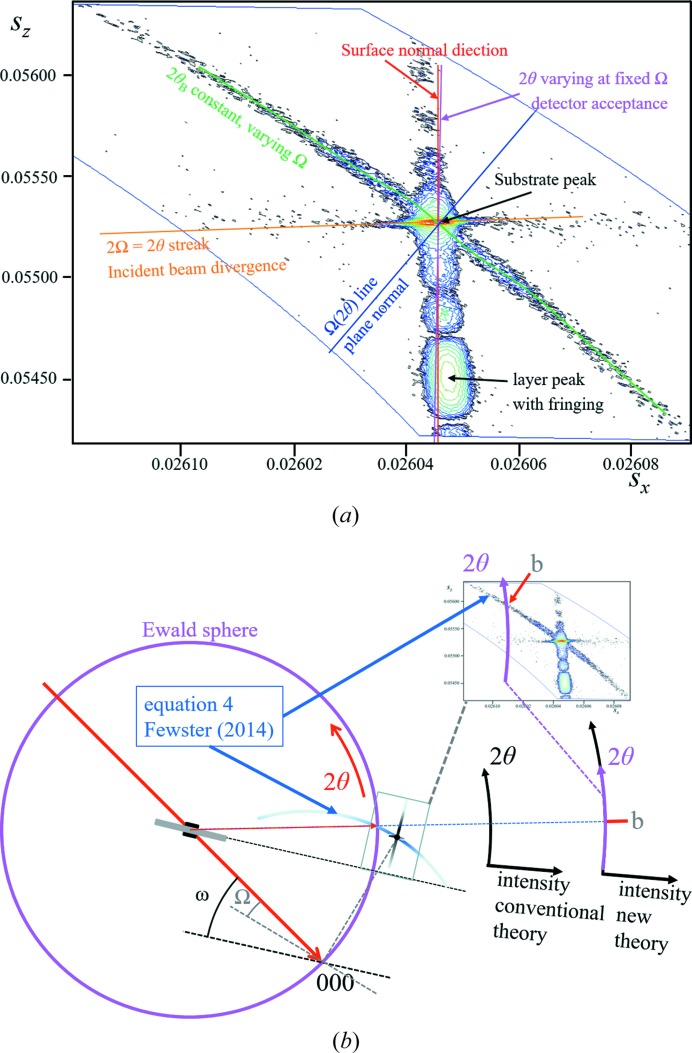
(*a*) The complex scattering from the 113 reflection from an Si (001) wafer, with an epitaxial layer of SiGe, obtained with a high-resolution diffractometer, courtesy of A. Kharchenko and J. Woitok. The fringing relates to the thickness of the SiGe layer, which can all be explained by conventional (dynamical) theory. The various features determined by the instrument and diffraction geometry are given in the figure. The streak of intensity at constant 2θ_B_ cannot be explained with conventional theory but is predicted by the new theory and explained in panel (*b*).

The presence of these 2θ_B_ peaks cannot be explained by conventional theory, since the reciprocal-lattice feature should just be a point with a truncation rod perpendicular to the surface. Within the new theory this is explained (Fig. 3 ▸ *c*). Another example is given in Fig. 4 ▸(*a*) where the shape effects are prominent, the instrument function contributions are clear and the only unexplained feature within the conventional theory description is the arc of intensity corresponding to 2θ_B_ for all measured Ω. The interpretation based on the Ewald sphere is given in Fig. 4 ▸(*b*). This 2θ_B_ streak is prominent because of the geometry and the careful alignment of the ‘reciprocal-lattice point’ to be in the plane normal to the rotation axis about 000 (Fig. 1 ▸). When the alignment is not so exact the streaking falls off more rapidly with angle (Fig. 18 in Appendix *A* [App appa]). Originally these streaks were dismissed as unexplained artefacts of the experiment, but they can all be explained by the new theory.

Since the structure-factor amplitude *F_hkl_* relates to the total scattering from the set of *hkl* crystal planes, then all the scattering associated with these planes should be included to determine it. If *F_hkl_* is assumed to only exist near the Bragg condition, then it will be an underestimate.

## 3. The diffraction from imperfect structures   

The conventional theory requires the crystal to be mosaic to account for data and to account for the suppression of dynamical effects. Crystals might be mosaic, but if this is a requirement then we are taking a risk in accepting that the kinematical approximation is valid. It is certainly reasonable to accept that crystal planes could be bent to accommodate point defects, dislocations and precipitates, but to assume that the mosaic blocks must be sufficiently small to suppress dynamical effects is difficult to accept.

**Figure 5:**
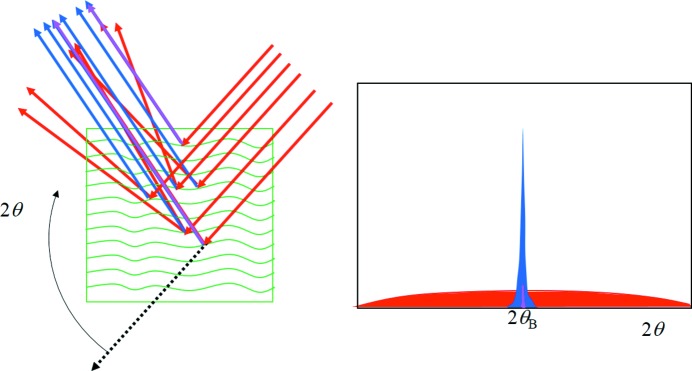
The formation of a diffraction peak in an imperfect crystal represented by undulating planes due to defects. The atomic planes scatter intensity in a direction defined by the crystal truncation rod, the specular angle 2θ (red), and towards the enhancement angle 2θ_B_ (blue). The specular peak is therefore broad, appearing as background, and the enhancement peak is sharp. Only for those scattering directions where the specular and enhancement contributions overlap (purple) will the Bragg condition and dynamical effects become important. For imperfect crystals, this overlap becomes a small fraction and *I* = |*F_hkl_*|^2^ is a reasonable assumption.

Considering that the crystal planes are curved but their separation is roughly constant, then the Bragg condition will be satisfied for regions on the crystal planes where the incident angle Ω = θ_B_. In a different region the Bragg condition is not satisfied but there will still be intensity at 2θ_B_ but it is weaker, as illustrated in Fig. 1 ▸. The different incident angle will also give rise to intensity at the specular position at 2θ somewhere on the crystal truncation rod. In another region, the scattering will also contribute again to 2θ_B_; however the contribution to the specular peak will move up or down the crystal truncation rod because of the different incident angle to the plane for that region. The specular peak therefore broadens and becomes less well defined, as regions of different curvature are probed, and forms a broad background peak (Fig. 5 ▸). Any specific location on the truncation rod will also be subject to the ‘enhancement effect’ (Fig. 1 ▸), but because a change in rotation is likely to bring another part of the crystal truncation rod closer to the optimum orientation (touching the conventional Ewald sphere) this effect will be masked. This description gives us an indication of how a polycrystalline sample diffracts by forming intensity consistently at 2θ_B_ and forming the background intensity. In the case of XFEL data the variability in the intensity at 2θ_B_ can be understood by the misorientation of the crystals from the Bragg condition, and since the incident intensity is so strong crystals do not have to be close to the Bragg condition to observe peaks at the Bragg angle.

## 4. The impact on data collection   

A single crystal diffracting in a random orientation is very unlikely to satisfy the Bragg condition for any reflections, but it will contain diffraction peaks that are very weak. This is observed at XFELs because of the very high incident-beam intensity. As the number of randomly orientated crystals increases more regions of the full diffraction pattern are explored which may contain a few Bragg peaks. The mean intensity of each diffraction feature becomes more representative of the full diffraction pattern. In polycrystalline diffraction all these individual patterns from each crystal are superimposed on each other to give the characteristic Debye–Scherrer rings with fluctuating intensity (Fewster & Andrew, 1993 ▸, 1999 ▸). This description explains the fluctuating intensity at XFELs. The diffraction pattern either from polycrystalline samples as the sum of contributions around the Debye–Scherrer rings or combined intensity contributions from individually indexed XFEL snapshots will stabilize when the full distribution of intensities has been explored and more contributions just confirm this (the central limit theorem). This is reached quite quickly in polycrystalline diffraction because of the large number of crystals in a typical experiment. In XFELs the intensity from each crystal is captured sequentially and will follow the same principle, making it possible to estimate the reliability in the intensities from the snapshot data. Structure analysis with single crystals collects the intensity near the Bragg condition for each reflection. This will not capture the full intensity distribution and could be subject to errors. In all cases the true mean of the intensity distribution will represent the intensity *I_hkl_*.

Since the intensity associated with a set of crystal planes is distributed, it is important to isolate the contribution associated with a specific *hkl* reflection. If data, as in single-crystal structure analysis, are only captured close to the Bragg condition, the ratio of the means of the full intensity distribution to those for the region of the capture will give a scale factor to obtain *I_hkl_*. In structure analysis the structure-factor *F_hkl_* amplitude is assumed not to be influenced by others, *i.e.* any overlap of their amplitudes from extended diffraction tails is ignored. The same assumption is made here, although an understanding of where the *I_hkl_* for one reflection ends and that from another begins along a truncation rod is helpful.

**Figure 6:**
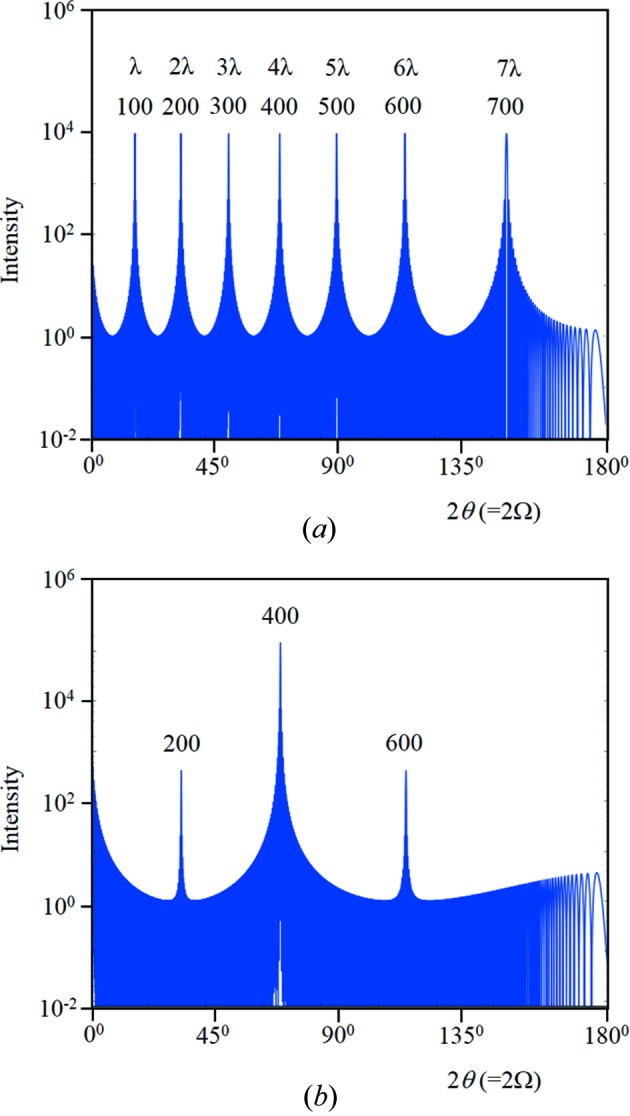
(*a*) This is the full calculated 2θ profile from *N* = 100 crystal planes (parallel to the surface plane) a distance *d* = 0.5654 nm apart and scattering strength of unity, *i.e.* there is one length scale (Cu *K*α_1_). The conventional diffraction peak indices are given as 100, 200, …, 700 and in the new theory these peaks are harmonics associated with the path lengths λ, 2λ, …, 7λ between adjacent planes. The intensity peak values correspond to *N* ^2^ and have not been modified by any factors. The truncation points used to define the range for individual structure factors occur in the dips between the peaks. (*b*) Inserting three more planes with the same periodicity but displaced by a fraction of this period at 0.25*d*, 0.5*d* and 0.75*d* and having scattering strengths of 0.9, 1.0 and 0.9, respectively, changes the profile dramatically. Individually each of these planes would reproduce the same profile as in (*a*), with the maxima modified by the scattering strength. However, when their amplitudes are combined, the peaks associated with path lengths of integer wavelengths *n* = 1, 3, 5, 7 result in their amplitudes cancelling – with *n* = 2, 6 the intensities are suppressed and the intensity for *n* = 4 is increased to (4*N*)^2^. This example profile is characteristic of a zincblende structure along the 〈100〉 direction.

The atomic plane specularly reflected intensity will exist for scattering angles 0 < 2θ < π, and each incident angle will also contribute intensity towards 2θ_B*n*_ in the range 0 < Ω < 2θ_B*n*_ (or π − 2θ < Ω < π/2 if 2θ_B*n*_ > π/2). This reflected intensity profile from a stack of crystal planes will have peaks whenever the path length is an integer number of wavelengths, *n*λ, the first when *n* = 0 which corresponds to *F*_000_ at 2θ = 0. The second peak occurs when *n* = 1 and occurs at 2θ = 2θ_B1_ which corresponds to the first-order Bragg condition in conventional theory. There will also be further peaks at *n* = 2, 3 *etc*., which are harmonics from path lengths of 2λ, 3λ *etc*. and do not reveal additional lengths in the structure (Fig. 6 ▸ *a*), *i.e.* this is the profile from a single set of periodic planes calculated using equation (16) in Appendix *B*3. The mid-positions between the peaks give a reasonable range that can be associated with each *F_hkl_*. It is reasonable to assume that the intensity contributions from the overlapping amplitudes at these mid-points will have little influence on the required *F_hkl_* values.

**Figure 7:**
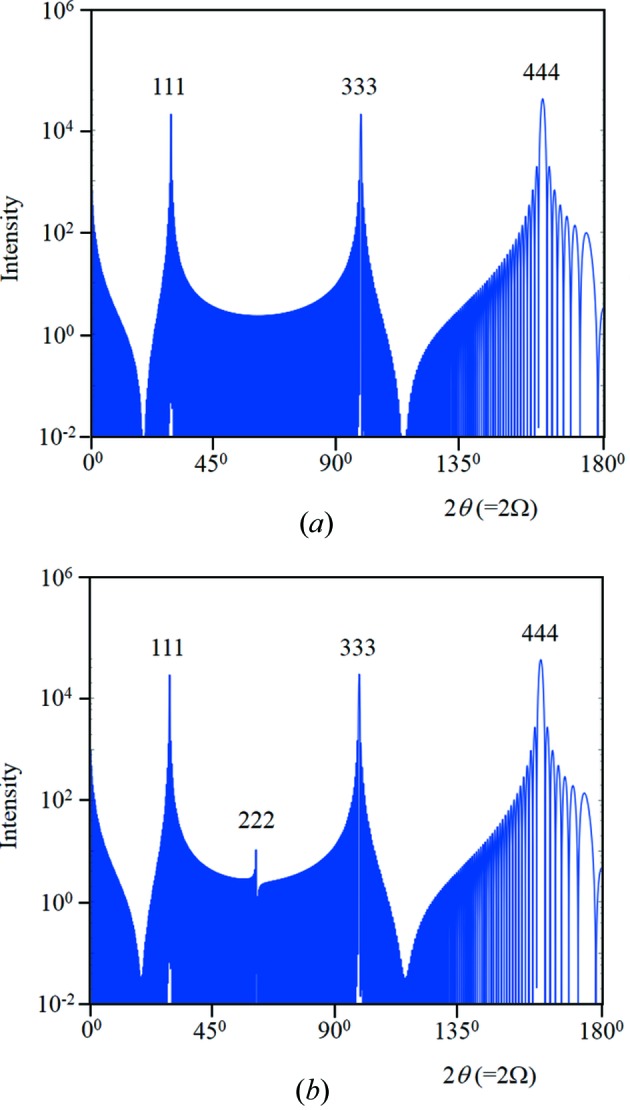
(*a*) This is the full calculated 2θ profile from *N* = 100 crystal planes a distance *d* = 0.31356 nm apart and scattering strength of unity, interlaced with planes at 0.75*d* also of strength unity. This is the pattern expected from Si along the 〈111〉 direction. (*b*) If the atoms are not confined to the planes but their electron density spreads symmetrically from these positions, then the 222 diffraction peak appears. This simulation includes additional scattering planes at 0.002*d*, 0.748*d*, 0.752*d* and 0.998*d*, with scattering strengths of 0.1.

Fig. 6 ▸(*a*) corresponds to a very simple structure. By adding more planes of atoms with the same periodicity and combining their amplitudes the resulting intensity is changed dramatically (Fig. 6 ▸ *b*) [equation (17) in Appendix *B*3]. By building the diffraction pattern in this way the electron density around the atoms can be included rather easily by adding thin layers representing the profile of the electron-density distribution. Thermal vibrations can be included in the same way to observe the change in the profile and the suppression of the maxima. For example, by taking a Si 〈111〉 orientated wafer and calculating the intensity along the truncation rod along 111 we obtain an intensity profile as in Fig. 7 ▸(*a*). This assumes the atomic planes have no thickness. If now the atoms have a spherical distribution of electrons, the lateral average of the electron density will be symmetrical either side of the planes and, by taking a thin layer of low electron density equidistant on both sides of each plane, the intensity profile is modified (Fig. 7 ▸ *b*). This example gives intensity at the 222 reflection that is ‘systematically absent’ in the conventional theory, which is often interpreted as asymmetry in the bonding (Bragg, 1921 ▸), but any analysis should include this diffraction effect. This has also been confirmed with dynamical theory, using Fresnel’s equations based on Holý & Fewster (2008 ▸) and Fewster (2015 ▸). Thermal vibrations will redistribute the electron density, reducing the peak intensities while maintaining the profile width and increasing the background (Guinier, 1963 ▸). This modifies the profiles along the specular direction, and the 2θ_B_ enhancement feature will be scaled in proportion. Further consideration is given in the calculations that follow later.

If the Bragg peaks are not on the same crystal truncation rod, then the interference described above will be weaker. In conventional theory there is no simple mechanism for them to overlap or interfere. However, for a wafer crystal with planes not accurately parallel to the surface plane with an epitaxial layer on top, some ‘wiggles’ in the overall truncation rod can be observed (Fig. 19 in Appendix *A* [App appa]). This can be interpreted as the interference of the crystal truncation rods of the substrate and layers, which is possible because of the significant overlap of the persistent intensity in 2θ for each contributor.

The following section presents a procedure for estimating the mean intensity to reveal the ‘structure factor’ for subsequent structural investigations.

## 5. The estimation of the intensities based on this concept   

The calculation of the resultant amplitudes in every direction for a very large number of atoms is currently impractical (even considering a crystal composed of structure-factor amplitudes). The approach by Fewster (2014 ▸) considers the scattering in terms of an ordered array of unit cells.

Ideally the calculation of perfect crystals should invoke dynamical theory; however the strength of these effects varies with the structure and crystal size. To illustrate the argument, it is assumed that the intensities all follow the kinematical approximation. If the relevant information is known (perfection, size, structure) then when the Bragg condition is encountered dynamical theory should be applied as in the work of Fewster (2014 ▸). These ‘perfect crystal’ examples should be considered in terms of nearly perfect crystals (where the dynamical effects are weak, *i.e.* some small plane curvature > the intrinsic diffraction width, *e.g.* ∼10 arcsec). It is also worth noting that a divergent incident beam will also add to the intensity but will not be at the Bragg condition and therefore not dynamical, which can be understood from the incident-beam divergence shown in Fig. 18.^4^ The total intensity *I*_total_ scattered from a set of crystal planes is therefore assumed to be ∝ |*F_hkl_*|^2^ and the total number of unit cells.

Not only are the contributions to |*F_hkl_*|^2^ dispersed, but the measurement method can lead to oversampling, which is most pronounced when the scattered waves leave the crystal plane at low angles such that the detector slit will capture a large range of sample tilts Δ*X* compared with higher exit angles:^5^

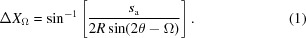

*R* is the sample-to-detector distance and *s*_a_ is the width of the axial slit. The angle of tilt *X* of the crystal plane has an axis parallel to the crystal plane and in the plane of the incident beam and the crystal plane normal (Fig. 20 in Appendix *A* [App appa]) (Fig. 10, Fewster, 2014 ▸). There will also be a spread in the incident-beam divergence, ΔΩ, and the Δ2θ acceptance at the detector, for a single measurement position *I*_2θΩ*X*_. The contributions in ΔΩ and Δ2θ are compressed into a smaller area at low and high scattering angles, requiring more steps to capture the intensity, which is compensated for with the term 1/sin 2θ. The proportion of the intensity incident on a plane varies as sin Ω_*X*_, provided it is totally immersed in the beam. Ω_*X*_ is the incident angle on a plane tilted by *X*. The calculation of the measured intensity within the bounds of 2θ, Ω and *X* of the experiment can then be written as 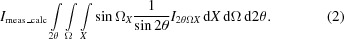
In single-crystal analysis the intensity is measured close to the Bragg position, where the incident-beam divergence is fixed, Ω ≃ θ, *X* ≃ 0 and the ‘out-of-diffraction-plane’ capture by the detector *s*_a_ is small compared with *R*. Equation (1) approximates to 1/sin θ, the sin Ω term cancels in equation (2) and the 1/sin 2θ term remains, as expected.

The intensity at a point *I*_2θΩ*X*_, for a series of *N* crystal planes of dimension (*L_x_* × *L_y_*) a distance *d* apart from a perfect crystal at an angle of 2θ to the incident-beam direction without measurement aberrations, is given by [equation (9), Fewster, 2014 ▸] 
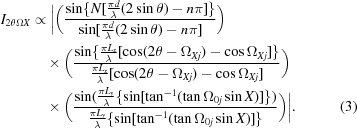
Ω_0*j*_ is the incident angle to the crystal plane along the *X* axis. The plane normal is tilted by *X* with respect to the detection position which is on the locus of the angle 2θ (Fig. 20 in Appendix *A* [App appa]). Ω_*Xj*_ is the actual incident angle to the plane at this tilted angle *X* [equation (6), Fewster 2014 ▸].

The first term can be considered as a shape transform, corresponding to a flat crystal wafer with the planes parallel to the surface, which has a maximum value at the Bragg angle. The width is the same as that given by Scherrer (1918 ▸) and can be modified for different shapes by using the average number of planes *N_hkl_*, *e.g.* Wilson (1963 ▸), Fewster (2015 ▸). The direction of this broadening was discussed earlier, which gives the full fringe pattern around the Bragg condition peak. The impact of not including all these fringes is not a problem provided the same dynamic range is assumed during the data collection; this is covered in §7. So as not to deviate too far from traditional methods, the structure factor is assumed to represent the full scattering for each reflection, *i.e.* *F_hkl_*. The integral of *I*_2θΩ*X*_ should therefore equate to |*F_hkl_*|^2^.

The second term gives the magnitude of the scattering reflected from a single-crystal plane (the Ewald sphere surface thickness profile), which has a maximum value of unity when 2θ = 2Ω_*Xj*_.^6^ The third term takes account of the combination of scattering points and their phases on an inclined plane, which also has a maximum value of unity. The derivation of the first two terms is given in Appendix *B*1 and the third in Fewster (2014 ▸). Equation (2) can be used to calculate the intensity in the measured region and the total intensity that could be measured from this set of crystal planes. The ratio makes it possible to scale the measured intensity and determine *I*_total_ and hence the structure factor: 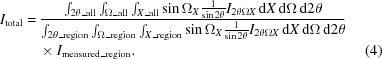
The denominator is set to have the same divergence, oversampling *etc*., as in the experiment to obtain *I*_meas_region_. The numerator represents the full intensity scattered above the crystal plane without oversampling and instrumental influences. The experimental measurement of this full intensity is impractical because by its very nature it will be entangled with the scattering from other crystal planes.

## 6. The nature of this calculation   

**Figure 8:**
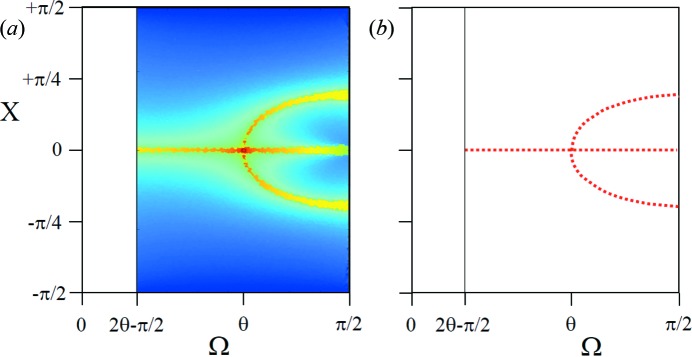
(*a*) The calculated distribution of intensity in Ω and *X* at a specific 2θ angle taken from Fewster (2014 ▸). This plot includes the oversampling and instrumental aberrations expected from a polycrystalline diffraction experiment, which is indicated by the smearing near the small exit angle when Ω ≃ π/2. (*b*) is a schematic of the regions of maximum, and dominating, intensity used in the calculations in this article, prior to any oversampling or other experimental influences.

The full calculation as in the work of Fewster (2014 ▸) is very computer intensive and a short-cut procedure is given here. As shown in Fig. 8 ▸(*a*) most of the intensity is along *X* = 0 and the ‘banana-shaped’ contribution where Ω_*Xj*_ = θ. Ω_*Xj*_ (< Ω_0*j*_) is the projected angle where *X* ≠ 0, such that as Ω_0*j*_ is increased there will be a point when Ω_*Xj*_ = θ and this occurs when 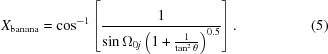
The *X* = 0 contribution corresponds to the term(2) in equation (3) with Ω_*Xj*_ = Ω_0*j*_, and term(3) = 1. The two ‘banana-shaped’ contributions above and below *X* = 0 correspond to term(3) in equation (3), with the locus of the maximum intensity, given in equation (5), occurring when term(2) = 1. To calculate the total intensity at a specific 2θ value that can be associated with the *structure factor*, the whole intensity over all of *X* and Ω needs to be estimated by independently determining the mean values of term(2) and term(3).

This has been achieved in Fig. 8 ▸(*a*) by sampling the intensity at random Ω and *X* values and superimposing the capture area of the experimental method. Much of the intensity in Fig. 8 ▸(*a*) is close to zero and ignoring this will not significantly alter the mean value; it can be compared with Fig. 8 ▸(*b*). It is necessary to choose a suitable capture length in *X*, which also allows all the analyses to be compared. The capture length Δ*X*_CL_ was set to 0.1°, so for example the intensity at *X* = 0, where Ω < θ, will be diluted by 0.1/180 *etc*. The number of Δ*X*_CL_ in π is labelled *Nu*. Any oversampling will therefore be digitized to this precision. The overall mean intensity that represents the structure factor will be represented by 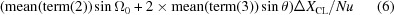
where *X* is set to *X*_banana_ or 0 if Ω < *θ* to evaluate term(3). The refractive index of the crystal defines the minimum and maximum Ω values, such that below a critical angle, which is set to 0.25° in these examples, the incident beam is totally externally reflected and conveys no structural information, and for very small exit angles the scattered intensity cannot emerge from the set of crystal planes.

To calculate the intensity at a specific 2θ value in a *polycrystalline diffraction* experiment, the data collection procedure must be considered. The axial slit at the detector of dimension *s*_a_ at a distance *R* from the sample captures a different range in *X* following equation (1). At very small exit angles from the crystal plane 2θ − Ω, the intensity associated with term(2) where *X* = 0 is captured almost regardless of the misorientation in *X* since Δ*X*_Ω_ → π/2, as sin(2θ − Ω) → *s*_a_/2*R*. The intensity scale factor at each Ω_0*j*_ position is therefore Δ*X*_Ω_/Δ*X*_CL_, whereas the intensity associated with the ‘bananas’ occurs at Ω = θ and is displaced around the ring and has a constant scale factor of Δ*X*_θ_/Δ*X*_CL_. The appropriate expression of the captured intensity is 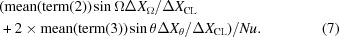
The ratio of equations (6) and (7) gives the scaling factor to extract the structure factor from a polycrystalline diffraction pattern.

In *serial crystallography*, a peak is identified and measured which can appear anywhere around the 2θ ring (the full ring corresponds to χ = 2π, where χ is the angle around the Debye–Scherrer ring, Fig. 20). The identification process will almost certainly isolate the peak from a crystal plane when *X* = 0. However the peak will have a finite width in *X* and therefore the intensity capture from isolating, indexing and measuring the integrated intensity will correspond to term(2) with a small angular or intensity range associated with term(3). The odd spots associated with the ‘bananas’ are most likely to be classified as artefacts and not measured. The intensity factor is similar to equation (7) except that term(3) is truncated; it is assumed that this is 1° in the following examples, which equates to *s*_a_/2*R* in equation (6) and with Ω = θ. The detection level for term(2) should also be considered, which will be covered in the examples below: 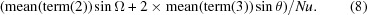
Since the intensity from *single crystals* is collected close to the Bragg condition, the mean intensity factor is the same as equation (8), except that the dispersed intensity is truncated close to the Bragg condition through background subtraction. The truncation of the intensity at different levels, as would be the case when measuring weak and strong diffraction peaks, will impact on the consequent analysis and is considered in the examples below.

## 7. The impact of the 2θ measurement range   

The above discussion relates to the intensity determined at one 2θ position, whereas any measurement will integrate the intensity around the diffraction peak. But how much of the intensity should be included either side of the peak along 2θ? To apply conventional structure determination methods a representative value for each of these individual peaks requires an estimate for the range over which the intensity should be isolated.

It is possible to assign an intensity associated with *F_hkl_*(*n*) over the range 2sin^−1^[(2*n* − 1)(λ/2)/2*d*] < 2θ < 2sin^−1^[(2*n* + 1)(λ/2)/2*d*] with the minimum of interference of adjacent peaks (see Fig. 6 ▸ *a*). The conventional theory assumes that the relevant intensity is associated with the length scale *d*/*n* = λ/(2sin θ) with *n* = 1, and it assumes there is just one peak associated with this length scale, which differs from this explanation.

Since it is in general impractical to capture intensity even over this limited 2θ range, the purpose here is to see whether it is possible to obtain a good estimate of the intensity associated with *F_hkl_*. The intensity is calculated within this range and compared with a truncated region close to the peak using equation (3) while keeping term(2) and term(3) constant. This has been calculated with peaks centred on 2θ_B_ values from 2° to 175°. The integrated intensity from the measurement range 2θ_B_ − Δ2θ to 2θ_B_ + Δ2θ has been compared with that obtained over this range to give the proportion of the intensity associated with the structure factor. Fortunately, this proportion is constant over the full range of 2θ_B_ values for all the crystal sizes studied from 1 to 250 µm, provided each diffraction peak is truncated at the same dynamic range of intensity. This ratio is 0.987 for intensity truncated at the 0.1% level, 0.96 at 1% and 0.886 at 10%. Clearly this must be considered when comparing intensities captured with different levels of truncation, which could for example lead to an underestimate of the structure factor for weak diffraction peaks without compensation.

As discussed earlier, thermal vibrations do redistribute the intensity along this 2θ line and their influence could be considered in two ways. Either simulate the profile and compare the impact of different ranges of capture, or more simply apply the conventional thermal parameter to the calculations here, which corresponds to a specific 2θ value. This normal application of the thermal parameters is a reasonable step since all other influences along the 2θ line have little effect on the intensity ratios, provided the truncation points are identical.

## 8. The influence of structural imperfections   

The intensity maps in Fig. 8 ▸ assume the sample is perfect; however this is unlikely to be the case and we need a method of generating the intensities from imperfect crystals.

**Figure 9:**
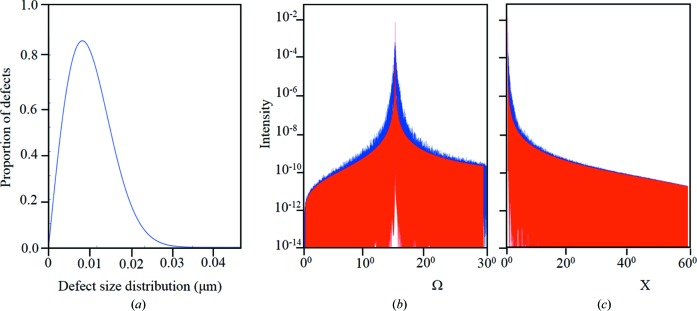
(*a*) The Weibull distribution, where the proportion of defects = (*k*/ λ)(*x*/ λ)^(*k*−1)^ exp[−(*x*/ λ)*^k^*] with parameters λ = 1 and *k* = 2. The basic curve is produced with *x* ranging from 0.001 to 3 µm, which is then scaled by (the mean defect size)/(proportion of defects) to give (*a*). This is used as the prior distribution for the dimension of the regions with varying orientations within an imperfect crystal. A Gaussian distribution is used as the prior distribution for the orientation of these regions. (*b*) and (*c*) show examples of the changes in the intensity profiles along Ω and *X*_banana_, perfect (red) and when defects are introduced (blue) for a 3 µm crystal, at a diffraction peak of 2θ = 30°. The distribution of distance between defects is given in (*a*) with an orientation FWHM of 0.58° (σ = 0.25°).

In the imperfect crystal calculation, the *L_x_* dimension is split into random lengths with different tilts to model the crystal planes that are not flat and each experiences a different incident angle. The equation and its derivation are given in Appendix *B*2. The mean of these lengths is defined by the defect separation, and the tilts are related to the size of the defects. The dimensions of these regions are drawn from a Weibull prior distribution (Fig. 9 ▸ *a*) for every intensity point evaluation to give a large variation of possible defect separations, and the tilts are drawn from a Gaussian distribution. It is assumed that the dimensions in the two orthogonal directions are the same, whereas the orthogonal tilts are uncorrelated. The Weibull distribution is very versatile and used in particle size and failure analysis distributions *etc.*; it gives a shape that could represent the distribution of defect separations.

In the examples below the Gaussian prior distribution was set to a range of full width at half-maximum (FWHM) values [0.0°, 0.58° and 1.16° (σ = 0.0°, 0.25° and 0.5°)], and the centre of the Weibull distributions were varied from 0.01, 0.1 and 1 µm for the 1, 3 and 10 µm imperfect crystals. The modification of the profile due to imperfections is illustrated in Figs. 9 ▸(*b*) and 9 ▸(*c*). Without a detailed analysis, these parameters are in general unknown, so either the microstructure should be estimated through measurement (as in the single-crystal analysis below) or as done here some judgement is made on various levels of imperfection and their impact on the intensities. These calculations put no restriction on the coherence length of the source or the regions over which the crystals are considered to scatter coherently. The contributions across a distorted plane are assumed to be in-phase, although if the coherence length of the source is limited then the regions or combinations of parts of regions can be added coherently and their intensities summed.

These calculations assume that the interplanar spacing is unchanged; however this is unlikely. Although the parameter space starts to explode, it is possible to explore the influence of fluctuations in *d* on the integrated intensity along 2θ. The formulae for these calculations are given in Appendix *B*3 with a high strain level of 1% (maximum random variation in *d* with depth). The ratio of the integrated intensities obtained close to the peak compared with those over the range representing the structure factor is constant for 0 < 2θ_B_ < π, and results in the same ratios as given in the previous section for the perfect structure.

## 9. Obtaining structure factors: examples   

This section describes various scenarios that indicate the importance of reconsidering the conventional approaches.

### 9.1. Comparing the full and short-cut calculations   

**Figure 10:**
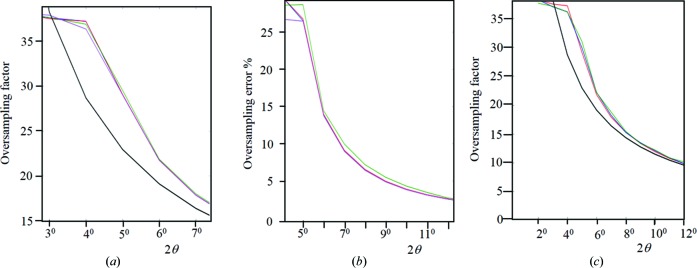
(*a*) shows the oversampling in a polycrystalline diffraction experiment based on the conventional theory, *i.e.* 1/sin θ, given in black, compared with the calculated oversampling based on the new theory using the simplified calculation. The overlapping profiles include 10, 3 and 1 µm perfect crystals in blue, red and green, respectively (Cu *K*α_1_) and 10 µm perfect crystals with Mo *K*α_1_ radiation, in magenta. (*b*) shows the percentage difference in the resulting extracted intensities between the conventional and new theory. The labelling of the profile colours is the same as in (*a*). (*c*) The influence of defects on the scaling factor: for a 3 µm perfect crystal (red), a crystal with a mean of 0.01 µm misorientated regions (based on the Weibull prior distribution and represents the separation between defects) with a FWHM of 0.58° (blue) and 1.16° (green) based on a Gaussian prior distribution. Different defect separations made no difference to the profile. The black profile is the 1/sin θ term. The presence of defects seems to have no influence on the scaling factor.

The first task is to ensure that the approximation compares with the calculations based on the full simulation, *i.e.* Fig. 8 ▸(*a*) compared with Fig. 8 ▸(*b*). The comparison is achieved by calculating the oversampling in polycrystalline diffraction as a function of 2θ for the two approaches. In Fig. 21 of Fewster (2014 ▸), the estimation of the oversampling compared with the conventional 1/sin θ dependence indicated a significant deviation at low 2θ angles and this is replicated in the ‘short-cut’ calculation, determined from equations (6) and (7). The oversampling in Fig. 21 of Fewster (2014 ▸) is normalized, because of the nature of the calculation, and only an approximate comparison made, and the effect is similar. The calculation in Fewster (2014 ▸) gave a slightly larger difference than contained here.^7^ These ratios are plotted in Fig. 10 ▸ as a function of 2θ, and correspond to the multiplication factor for *I*_meas_calc_ in equation (4) for a polycrystalline diffraction experiment with 10 µm perfect crystals and a Soller slit of 0.04 radians. Both calculations indicate that the intensities in polycrystalline diffraction lead to a greater oversampling of the intensity for low 2θ peaks, *i.e.* the conventional ‘1/sin θ’ term is too small and will suggest that the structure factors in this region are overestimated. This short-cut calculation is a good approximation for estimating the relationship between the measured data and the structure factor. This deviation compared with the conventional 1/sin θ will be most pronounced in analysing polycrystalline materials with large lattice parameters, *e.g.* proteins.

### 9.2. Polycrystalline diffraction   

The calculation in Fewster (2014 ▸) and that described in the preceding paragraph is for perfect crystals, whilst in this section the influence of wavelength, crystal size and imperfections is explored. The same calculation above is repeated for Mo *K*α_1_ radiation, which is approximately half the wavelength of Cu *K*α_1_, and the profile is unchanged (Figs. 10 ▸ *a* and 10 ▸ *b*). This shows that harder radiations, which shift the diffraction peaks to lower 2θ values, will create a larger deviation in the structure factors compared with the conventional 1/sin θ term.

The parameter space in combining crystal size and defects is large, and the following analysis provides some broad conclusions. The scaling parameters have been calculated for three crystal sizes (1, 3 and 10 µm) and a range of imperfections drawn from prior distributions based on crystal regions of 0.01, 0.1 and 1 µm, and separated by mean orientations of 0.58° and 1.16°. Fig. 10 ▸(*c*) shows the systematic error in the intensities for deriving the structure factors compared with the conventional 1/sin θ function, for 3 µm crystals with both angle orientations for the 0.01 µm typical defect separation. It is reassuring that the systematic error is consistent and is independent of these parameters.

The reliability will depend on the number of crystals contributing to the diffraction pattern, and the conclusions are the same as those given in the following section on serial crystallography.

### 9.3. Serial crystallography   

As well as the influence of different sizes and imperfection levels, it would be useful to know how many crystals are required to obtain a reliable intensity estimate for each *F_hkl_* value.

**Figure 11:**
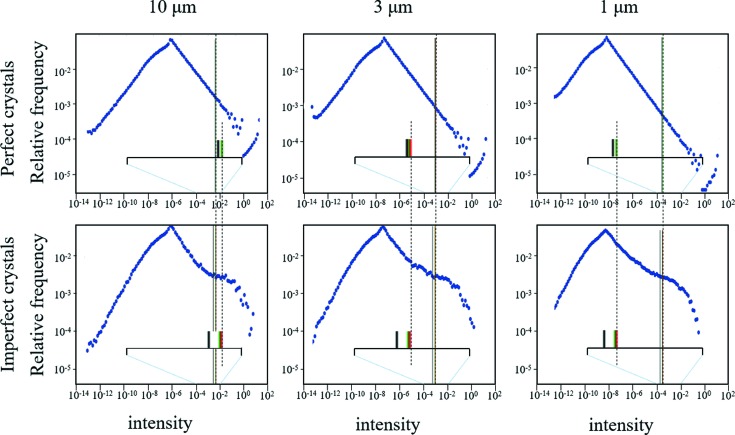
These are the calculated distributions of the integrated intensities at 2θ_B*n*_ = 30° with Cu *K*α_1_, for a set of crystal planes in random orientations, for three crystal sizes. The maximum intensity (the rightmost data point in each histogram) corresponds to the Bragg condition, Ω = θ_B*n*_. The mean intensity gives a good representative parameter for the total scattering from these planes. The ‘true mean’ (red line) associated with the total scattering is approximately constant for perfect and imperfect crystals (σ = 0.25°) for all crystal sizes (∼3% fall in intensity). If the intensity is just captured near the peak over two orders of magnitude the mean values fall, and the structure factor is underestimated by 2% for perfect crystals and 3% in imperfect crystals. If the data are only captured over one order of magnitude, then the structure factor is underestimated by 5% for perfect crystals and by 20% for imperfect crystals (the green line in the figure). This calculation includes 5 000 000 orientations to ensure the profile is fully converged.

The influence of crystal size and imperfections changes the distribution of the intensities measured, as can be seen in Fig. 11 ▸. The mean value of the total scattered intensity for imperfect crystals (σ = 0.25°, 0.01 µm mean distance between defects) is 3% below that for a perfect crystal, *i.e.* the mean of the total intensity is very similar for imperfect and perfect crystals. But if the intensity is only measured close to the peak, *e.g.* to within 1% of the peak intensity in a perfect crystal, ∼4% of the intensity will be missing and ∼6% for imperfect crystals (for all these crystal sizes with respect to the mean). If a reflection is weak and can only be measured to within ∼10% of its peak value, the missing intensity amounts to ∼11% for perfect crystals and ∼37% for imperfect crystals. Thus, measuring the intensity to within two orders of magnitude will reduce the structure factor by ∼3%, or if the intensity is only measured to within one order this results in a ∼20% reduction. Fortunately, these proportions are practically constant over 2θ, but combining weak and strong reflections needs more thought, which is considered in the next section.

**Figure 12:**
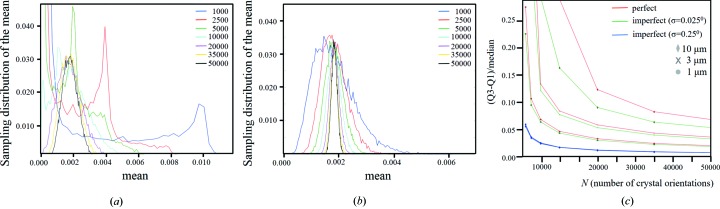
(*a*) The mean values of integrated intensity calculated for a 3 µm perfect crystal at 2θ = 30°, from a crystal in a random orientation, for increasing numbers of contributions. These curves give the spread in the calculated estimated mean values. As the number of contributions increases the spread in the estimated mean narrows and centres on the true mean value, but even for 50 000 contributions the reliability in measuring the mean is poor. (*b*) This is case for an imperfect 3 µm crystals (σ = 0.25°), where the mean is much better defined because of the suppression of the peak and increase in the dispersed intensity. (*c*) When the interquartile range (Q3–Q1) over the median decreases the confidence level increases and the true mean intensity becomes reliable. This plot shows that this ratio reduces with the number of crystal orientations being explored for three sizes of perfect and imperfect crystals, for 2θ_B*n*_ = 30°. Clearly, as the defect level increases and the crystal size reduces, fewer numbers of crystals are needed to determine a reliable intensity value.

This distribution makes it possible to estimate the number of crystals required in serial crystallography to achieve a reliable intensity value (Fig. 12 ▸). Suppose there are *N* crystals in an experiment, then the sum of all the measured intensities for a given *hkl* divided by *N* will give an ‘estimated true mean’ value; however the reliability of this estimate may not be a good representation when compared with that from a very large number of crystals, as presented in Fig. 11 ▸. The reliability of this ‘estimated true mean’ can be judged by randomly sampling *N* crystal orientations from this large population of intensity values and calculating an ‘estimated true mean’ value to compare with the ‘true mean’. Repeating random sampling many times (20 000 in this example) will produce a distribution of the ‘estimated true mean’ from *N* crystals for comparison with the ‘true mean’ (Fig. 12 ▸ *a*). This distribution gives an estimate of the reliability in obtaining the ‘true mean’ from a single measurement with *N* crystals and therefore the reliability in the structure factor. This distribution clearly shows that many more perfect crystals are required to achieve any reliability in the structure factor, compared with imperfect crystals (Fig. 12 ▸ *b*).

This analysis has been applied to different sizes of perfect and imperfect crystals. The metric used is the median and interquartile range, which are more appropriate than the mean and standard deviation (σ) because these distributions are not necessarily Gaussian except at higher *N*. When the mean and median converge the interquartile range represents −0.67σ to +0.67σ. Fig. 12 ▸(*c*) gives the interquartile range divided by the median as a function of the number of crystal orientations for 10, 3 and 1 µm crystals. When this ratio has a low value then the confidence in the data is high. This shows that small imperfect crystals will give more stable intensities, but of course imperfect crystals will form weaker peaks and the question of dynamic range of intensity becomes important.

This may not be the only intensity measurement error and it will need to be combined with source intensity fluctuations and detector response *etc*. to estimate the reliability in the evaluated structure factor.

### 9.4. Single-crystal analysis   

**Figure 13:**
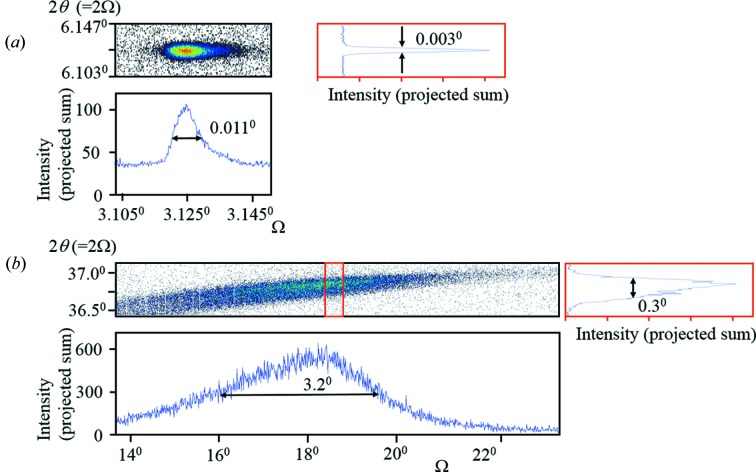
High-resolution diffraction space maps of different diffraction peaks from a high-quality (*a*) and poor-quality (*b*) lysozyme crystal each of a few hundred micrometres (crystals courtesy of E. H. Snell in 1996). These maps obtained with the multiple-crystal diffractometer, described in Fewster (1989 ▸, 2015 ▸), can be used to estimate the crystal quality from the FWHM in Ω to calculate an approximate intensity distribution (Fig. 14 ▸). The estimation will make assumptions regarding the number and therefore dimensions of the regions of similar curvature; however, as discussed in the text, if the regions scatter with a coherent relationship the size broadening effects will have less impact on the FWHM and the curvature will dominate, *i.e.* the number of regions in the calculation is not critical. With this assumption, the crystal plane curvature is equivalent to 3.2° in (*b*) and 0.01° in (*a*). The inclined diffraction spot in (*b*) results from curvature of the crystal planes normal to the figure resulting in a projection, revealed by three-dimensional reciprocal-space mapping (Fewster, 2015 ▸). This tilting in two dimensions also makes this map projection unreliable for obtaining precise 2θ values.

**Figure 14:**
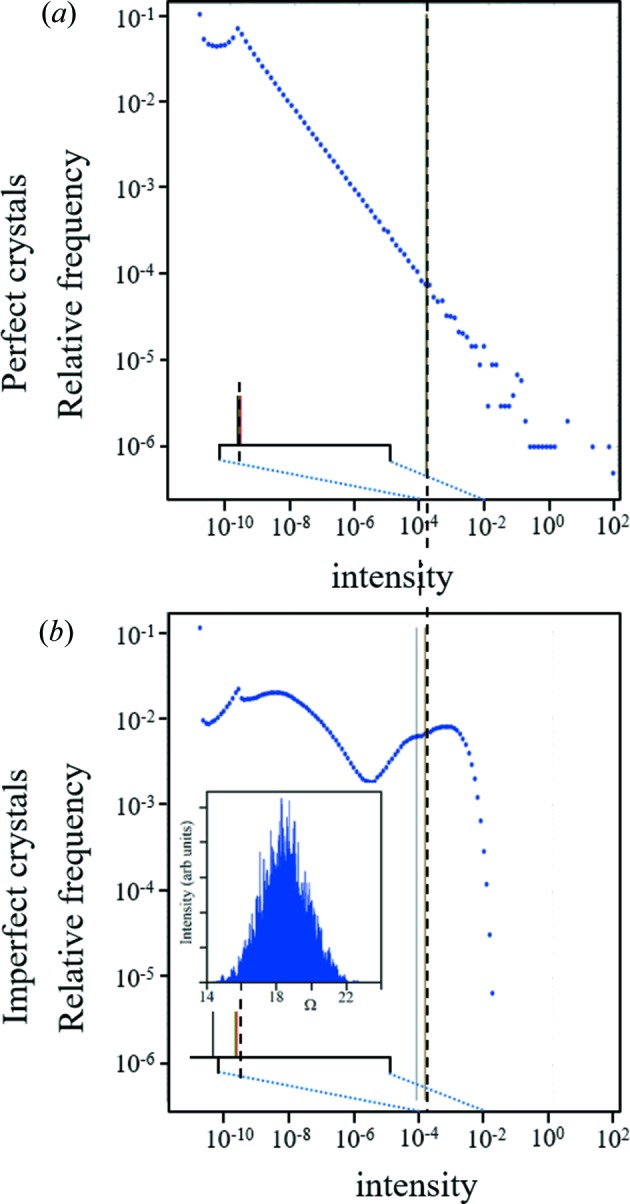
(*a*) The calculated intensity distribution assuming the crystal is a perfect lysozyme crystal, with a dimension of 250 µm. (*b*) The calculated intensity distribution based on the Ω profile width for the lysozyme crystal given in Fig. 12 ▸(*b*). The inset gives the Ω profile generated from these calculations. The mean value has fallen by 18% for the imperfect crystal compared with the perfect crystal, so this should be considered. The mean value of capturing the intensity over two orders of magnitude and the true mean result in a very small loss of 1.8% in structure factor, but only capturing intensity over one order of magnitude results in a structure factor loss of 5% for perfect crystals and 27% for imperfect crystals. 2θ_B*n*_ = 36.75°. Therefore, both the crystal quality and dynamic range of the measurement are important to obtain good structure factors.

**Figure 15:**
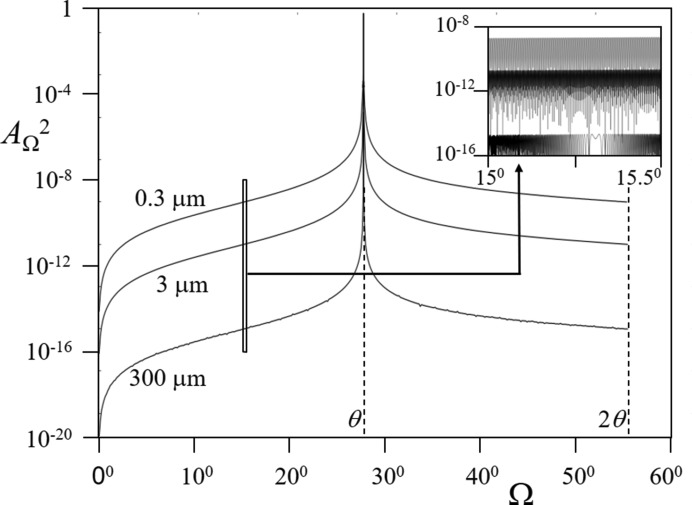
The variation in the specular (amplitude)^2^ for several crystal plane dimensions as the incident angle Ω is changed from the optimum value at θ [from Fig. 4(*b*), Fewster, 2014 ▸]. This profile is for perfect crystals (with full coherence across the plane) and can be considered as the thickness profile of the Ewald sphere, equation (10) in Appendix *B* [App appb].

**Figure 16:**
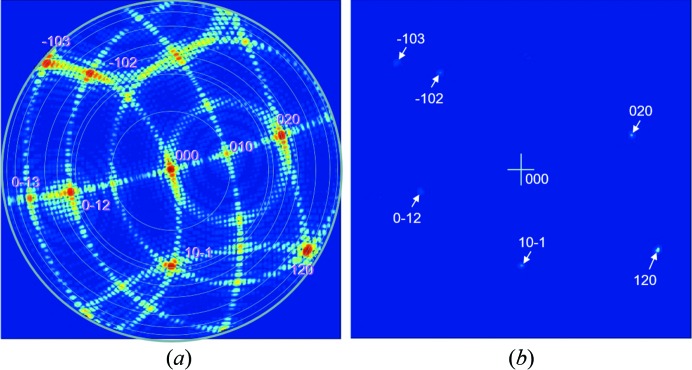
The simulation of the diffraction pattern from a three-dimensional array of point scatterers with dimensions 40 × 39 × 40 nm with point separations of 2 × 3 × 4 nm using a wavelength of 1.54 nm. The whole pattern is revealed in a logarithmic plot (*a*). When plotted on a linear scale (*b*) there are six ‘peaks’ observed. This is very characteristic of data from XFELs. Diffraction based on the conventional theory would reveal nothing in this arbitrary orientation (these are not in the Bragg condition). The central peak in (*a*) is the direct beam and is removed from the linear plot in (*b*), to reveal the other peaks with linear scaling. The plots are displayed on a radius of 2θ out to a maximum of 90°. The peaks can be indexed based on their 2θ_B_ values and the restriction 0 < Ω < 2θ_B_, yet their intensities vary significantly indicating that the reciprocal-lattice points cannot all be close to their Bragg conditions. It can be seen in (*b*) that on a linear scale that peak intensities <∼1% of the most intense peak are not observed.

**Figure 17:**
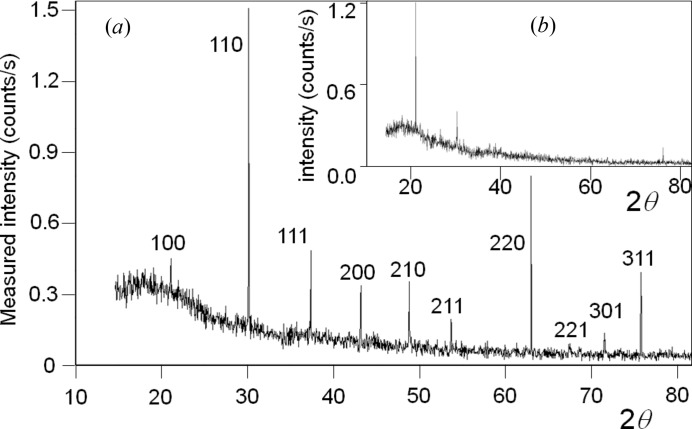
[Fig. 5 in Fewster (2014 ▸)]: (*a*) the scattering pattern from ∼120 crystals (or if perfectly packed 300 crystals) isolated with a 3.5 µm incident beam that perpendicularly intersects a 1 mm-wide single layer of crystals of LaB_6_ with sizes varying from 2 to 5 µm. (*b*) gives the profile with ∼30 crystallites or if perfectly packed 75 crystallites (3.5 µm × 0.25 mm sample size), where not all the reflections are clearly resolved as in the larger sample size. The data were collected with a 0.01° divergent Cu *K*α_1_ beam from a 1.8 kW X-ray laboratory source in 35 min. The samples were stationary throughout, so the incident beam only explored one orientation from each crystal. The peaks are narrow and occur at the correct Bragg angles, and correspond to the interpretation where each crystal contributes intensity as in Fig. 1 ▸.

**Figure 18:**
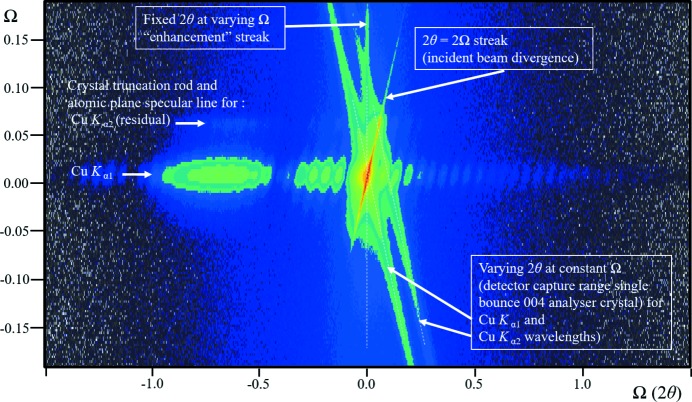
(*a*) A diffraction space map close to the 004 reflection from an InGaAs structure grown epitaxially onto a GaAs substrate. The data were collected with the beam selection diffractometer (Fewster, 2004 ▸), with a single reflection 004 analyser crystal (stepping in Ω followed by a scan with movements in Ω and 2θ maintaining a 1:2 ratio). The strong fringing is associated with the layer structure (the shape transform) and occurs along the crystal surface normal. The streak where 2θ = 2Ω corresponds to the incident-beam divergence and the streak along 2θ for a constant Ω value corresponds to the detector acceptance range (in this case the diffraction profile of the analyser crystal). The remaining streak at constant 2θ_B_ for varying Ω values is the ‘enhancement’ peak for the substrate (as in Fig. 4 ▸ *b*).

**Figure 19:**
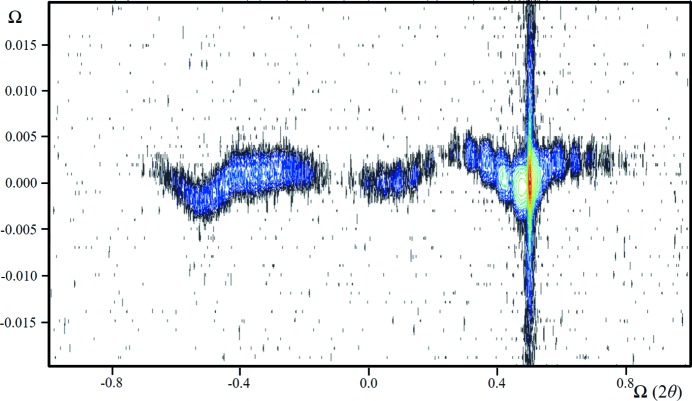
The 004 diffraction space map expanded normal to the crystal truncation rod to emphasize the wavy streak of the 80 Å In_0.15_Ga_0.85_As quantum well, buried in a complex AlGaAs/GaAs structure. The other dominant feature is the streak along 2θ_B_. When the data were projected along 2θ_B_, the resultant profile fitted precisely with the simulation based on dynamical theory. Collecting data with a high-resolution diffractometer without an analyser (a rocking curve) gave small fringe displacements with a broadened base to the substrate peak (a commonly observed feature), whereas a single scan along the crystal truncation rod gave regions of missing intensity.

**Figure 20:**
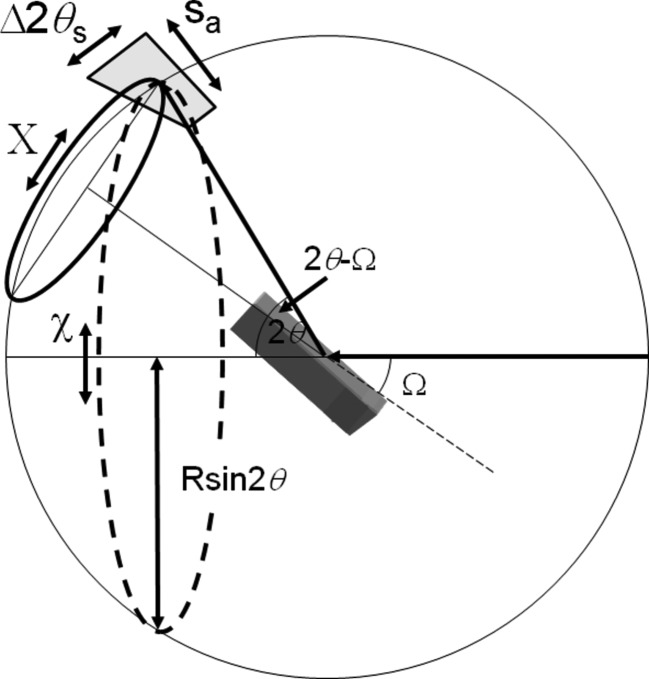
The relationship between the crystal plane, the tilt axis and the detector slit.

The typical crystal dimensions are much larger for this type of analysis, and this gives the opportunity to evaluate the perfection and feed this into the analysis. An example is given for two ∼250 µm-sized lysozyme crystals of good and poor quality (Figs. 13 ▸ *a* and 13 ▸ *b*) which have the equivalent FWHM values of 0.011 and 3.2°, respectively. Intensity distributions were calculated at one of these 2θ_B_ values (Figs. 14 ▸ *a* and 14 ▸ *b*) using the width for the poorer-quality crystal (Fig. 13 ▸ *b*), and assuming a perfect crystal, like that given in Fig. 11 ▸. There are several conclusions from this analysis. The ‘true’ mean intensity has dropped by 18% for the imperfect crystal compared with the perfect crystal, and an insufficient dynamic range of intensity captured will give significant errors in the structure factors. Further details are given in the figure captions. The large difference in the mean scattered intensity for the perfect and imperfect crystal could possibly be explained by the inadequacy of the assumption that the scattering is confined to the regions given in Fig. 8 ▸(*b*) rather than Fig. 8 ▸(*a*). It should also be remembered that this calculation does not include all the scattering, *e.g.* below the crystal plane which becomes more significant with high levels of imperfection.

The problem of weak intensities can be resolved by either truncating the intensity at an equivalent angular range for all diffraction peaks, then removing the background and accepting that the data will be noisy and limit the structural detail, or modelling the profile based on the shape of the stronger peaks and estimating the values of all the peaks over the same dynamic range. This latter approach obviously has strengths in extending the measurement of peak intensities with noisy data, and perhaps extending the resolution of the structural model.

## 10. Concluding remarks   

This new theory does have an impact on the assignment of intensity to the structure factors in polycrystalline diffraction, serial crystallography and single-crystal analysis.

Polycrystalline diffraction: the summation of the intensity at a specific 2θ will allow a good representation of the structure factor but it is oversampled. The oversampling differs from the conventional 1/sin θ term, which becomes more extreme for 2θ values below ∼12°. This deviation is independent of the wavelength used or the degree of crystal perfection. The difference in the intensity scaling factor is >25% at 5° and falls to 1% at 25° in the new formulation compared with the conventional term. The structure factor at low 2θ derived from the measured data based on the conventional theory is therefore too large.

Serial crystallography: the variable intensity from snapshots can be explained as a diffraction effect and each crystal will form several diffraction peaks at a single orientation. The intensity captured close to the expected peak position will give a highly asymmetric distribution that is a function of the crystal size and quality. When sufficient crystals have been analysed the mean intensity is Gaussian distributed and the uncertainty is defined (central limit theorem). The mean intensity should give a good direct relationship with the structure factor. The most reliable mean values occur for small imperfect crystals.

Single-crystal analysis: for large crystals typically used in this analysis the proportion of the uncaptured distributed intensity is significant for high levels of imperfection. This is also the case for smaller crystals. The most reliable analyses should model the intensity distribution to obtain a scaling factor for accurate structure-factor determination. The missing intensity from weak diffraction peaks should be compensated since the errors associated with this could be considerable.

This theory has an impact on how the diffraction pattern is formed, including the background, the scattering from imperfect materials, accounting for the 222 diffraction found in diamond-type structures *etc*. This theory can make the analysis easier, *e.g.* removing many of the corrections applied in serial crystallography, providing a simple way of probing the electron density and making a clearer judgement on the reliability of results from randomly orientated crystal data. The peaks in a diffraction pattern are formed from *n*λ path length changes and not from fractions of the repeat distances (*d*/*n*) as usually assumed in the conventional theory; it is therefore the amplitudes of the harmonics associated with each set of contributing planes that give rise to the peak intensity observed. These simulations indicate the limit on the validity of the structure factor for modelling structures.

## Footnotes

1 The parallel component of the electric field must be continuous across the crystal surface boundary and this defines where the scattered wave will be observed for the orientations away from the Bragg condition. This results in crystal truncation rods normal to the surface plane of a large crystal wafer regardless of the inclination of the plane to the surface. The shape of the profile can be calculated with dynamical theory.

2 This is the general case and does not include the Renninger (1937 ▸) effect: different Bragg diffracted intensities combining to form intensity at a systematically absent position (*Umweganregung*) and the suppression of diffracted intensity from this effect (*Aufhellung*).

3 If the intrinsic diffraction widths are compared, then this factor is closer to 40×.

4 A change in the incident-beam direction changes its own 2θ = 0 position.

5 This can be understood from powder diffraction rings, where the same lateral slit size will capture a bigger angular section of the ring at low angles of exit than at high angles. Conventional powder diffractometry applies a 1/sin θ_B_ term and assumes that all the intensity is from the Bragg condition.

6 This is the specular condition and corresponds to the 2θ angle associated with the Ω angle that gives rise to it (see footnote 1^1^). At the Bragg condition 2θ = 2Ω, and when the crystal planes are parallel to the surface. Later it will be seen that the profile along 2θ has little influence on the analysis.

7 This approach is more transparent and a much more practical route to obtaining better estimates of the intensities. The present calculations take seconds or minutes depending on the defect description, whereas the original calculations took weeks of computer time assuming the crystals were perfect. There is a small error in Fewster (2014 ▸), which may have led to an overestimate of the oversampling but the impact, if any, is difficult to assess without excessive amounts of work. The correction of the error would bring the oversampling closer to the results presented here, so it is reasonable to assume that the present calculations are closer to the truth and are much easier to check. The full calculations are fraught with difficulties (faster computers and better coding would help) so are not repeated, but importantly the nature and sense of the oversampling compare well in both calculation approaches.

## Appendix A Further diagrams and experimental evidence

Further explanatory diagrams and experimental evidence are given in Figs. 15 ▸ ▸ ▸ ▸ ▸–20 ▸.

## Appendix B The amplitude calculation method

This appendix gives the derivation of the equations used in the above calculations, both for perfect and imperfect crystals.

### b1. The intensity from a perfect sample

The amplitude *A* scattered from a row of *M* + 1 atoms is given by

where δ is the phase difference of the scattered waves from adjacent atoms. The amplitude at the centre of this row is given by multiplying this through by exp(*M*δ/2), giving rise to terms that can be paired as [exp(−*m*δ + exp(+*m*δ)], which is simply 2cos(*m*δ). The phase difference is given by δ = 2π/λ (path difference). If the planar incident wave makes an angle Ω to this row of atoms and the amplitude is monitored at an angle of 2θ to this incident-wave direction, then the path difference between neighbours *x* distance apart is *x*[cos(2θ − Ω) − cosΩ] (Fewster, 2014 ▸). The amplitude *A* from a single plane is the integral sum of these individual amplitudes over the length of the plane *Mx* (= *L_x_*), and is given by

where δ′ = δ/*x* = [cos(2θ − Ω) − cosΩ]. The amplitudes *a* do not necessarily have to be just from an atom, but can be from a group of atoms, *e.g.* a molecule or unit cell, but they must have the same amplitude and be at a regular repeat distance. The amplitude sum of numerous parallel rows will form the resultant for a plane.

Since there are many planes, *N*, that are equally spaced with each experiencing an amplitude *A* [equation (10)], their combined amplitude *A_N_* = *A* + *A*exp(*i*Φ) + *A*exp(*i*2Φ) + *A*exp(*i*3Φ) + … + *A*exp[*i*(*N*−1)Φ], where Φ is the phase change from plane to plane. This amplitude *A_N_* can be represented as a geometric series which is given by

The multiplicative exponent term simply moves the reference phase point from the top layer to the middle of the stack of *N* crystal planes for all the scattered waves. This distance compared with that to the detector is negligible in this plane-wave assumption, and therefore does not change the amplitude. The phase difference between the scattering from parallel crystal planes is zero when the path length between pairs of scatterers across adjacent planes is λ, which occurs when 2θ = 2θ_B*n*_ regardless of the incident angle Ω (Fewster, 2016 ▸). Therefore the maximum intensity occurs when the phase difference Φ = (2π/λ)(2*d*sin θ) − *n*(2π) is zero where *d* is the interplanar spacing and *n* is an integer, which corresponds to the number of wavelengths in the path difference; for example with *n* = 2, 3 *etc*., there will be additional enhancement peaks. The overall amplitude is the same as equation (5) in Fewster (2014 ▸). This amplitude term represents a significant contribution to the intensity, but should include contributions from inclined planes to the data collection point; equation (3) and derivation of the term(3) are given in Fewster (2014 ▸).

Equation (11) has two terms, giving rise to two peaks, *i.e.* when δ′ = 0 and when Φ = 0 which refer to the atomic plane specular peak and the enhancement peak, respectively.

In general crystals are not perfect, and this modifies the above formulae.

### b2. The intensity from an imperfect sample

As discussed by Fewster (2016 ▸) the peak intensity at 2θ_B*n*_ is weaker in imperfect crystals because the specular contribution is broad and therefore only a small proportion can overlap the enhancement peak for a specific wavelength and *d* spacing. The maximum intensity of the broadened specular peak from a slightly imperfect crystal is less than the enhancement peak. This smaller overlap leads to a weakening of the enhancement peak, because fewer contributions are in-phase and the intensity is increased elsewhere. Consider the effect of an imperfect crystal by applying the same approach as above. We would expect a bent crystal plane to experience different incident angles across it as well as a spread in the separation between the planes.

The amplitude from a series of *m* regions that have roughly flat crystal planes containing *M_m_* scattering centres is given by

Each region compares with equation (9) and the exponential terms outside the brackets bring the phases into registry; in this case they are relative to the middle of the first region. Each region can then be simplified as in equations (9) to (10) and combined with the influence of the number of planes, equation (11), which is included for each region:

where

The amplitude incident on each region is in proportion to the size of the region *l_x_* with respect to the whole length *L_x_*. The phase difference δ*_j_* between individual scattering centres in region *j* is (2π/λ){*x*[cos(2θ − Ω*_j_*) − cosΩ*_j_*]}, where Ω*_j_* is the local incident angle. The exp(*M*δ/2) term places the reference phase to the start of the region. Equation (13) is identical to equation (11) if the crystal is flat, since Ω*_j_* and *d_j_* would be the same for all regions. To include large numbers of defects, or to probe large regions, the phase can be averaged by making *M* random. The same methodology is used for the inclined planes.

If we assume that the crystal is not strained and the number of planes in each region is the same, then the calculation is simplified [equation (15) is the same for all regions]. It is clear with this approach that the level of complexity can be extended (including crystal shape as well as distortions, the influence of defects that define the relationship between Ω and *d* *etc*.) to the point where the whole pattern is a summation of all the point scatterers, but the latter is impractical to compute even for small crystals. Our main concern here is to estimate the intensity, *i.e.* what is measured close to a diffraction peak and what fraction of the total scattering power this represents.

Equation (13) can therefore mimic the broad specular peak and the sharp enhancement peak observed in Fig. 5 ▸.

### b3. The calculation of the profiles in Figs. 6 ▸ and 7 ▸

The amplitude specularly reflected from *N* planes separated by *d* is given in equation (15); however, in this case we drop the *n*π, because we are not trying to isolate an individual peak:

Whenever 2*d*sin θ/λ is integer then this term is at a maximum. Calculating the intensity (*A*_2θ_)^2^ over the range 0 < θ < π/2 will produce a series of peaks as in Fig. 6 ▸(*a*), depending on the value of *d*.

Inserting more sets of *N* planes interlaced with the original set will require the phase reference point to be made coincident with the original; if this is related to the first plane then this will be given by

The parameter *a* is the relative magnitude of the amplitude for the planes with respect to *a*_0_. This will reproduce the profiles in Figs. 6 ▸(*b*), 7 ▸(*a*) and 7 ▸(*b*).

This approach makes it possible to expand the modelling to strain by splitting the numbers of planes into smaller regions with common *d* values and relating the phase reference point of succeeding numbers of planes to that of the end of the previous set of planes. The phase term would be exp(*i*4π*d_j_*_−1_ *N_j_*_−1_sin θ/λ). This was the formula used for determining the influence of strain on the proportion of the structure factor measured with respect to that from the whole range.
